# Evaluation of Six Commercial and Noncommercial Colistin Resistance Diagnostics

**DOI:** 10.1002/mbo3.70029

**Published:** 2025-06-29

**Authors:** Tumisho Mmatumelo Seipei Leshaba, Masego Mmatli, Nontombi Marylucy Mbelle, John Osei Sekyere

**Affiliations:** ^1^ Department of Medical Microbiology, School of Medicine University of Pretoria Pretoria South Africa; ^2^ Institute of Biomarker Research, Medical Diagnostic Laboratories, Genesis Biotechnology Group Hamilton New Jersey USA

**Keywords:** antibiotic resistance, colistin resistance, diagnostic assays, *Enterobacterales*, molecular tests, polymyxins

## Abstract

Resistance to colistin, a last‐reserve antibiotic used for treating drug‐resistant infections, is rising globally. We compared six commercial and in‐house diagnostics—ComASP colistin, CHROMagar COL‐APSE, rapid polymyxin NP (Nordmann/Poirel) test, Sensititre, MicroScan, and Vitek 2—against ISO‐standard broth microdilution (BMD) using 142 Gram‐negative isolates. The isolates (*Enterobacterales* = 110, *Acinetobacter baumannii* = 21, *Pseudomonas aeruginosa* = 11) underwent BMD and conventional multiplex PCR screening for *mcr‐1*–*mcr‐5*. Sensitivity, specificity, categorical agreement (CA), major error (ME), and very major error (VME) were calculated for each test. Vitek 2 and sensititre yielded the highest CA (≥ 98%) and the lowest VME (≤ 0.0%) across taxa. ComASP showed excellent performance for *A. baumannii* (100% sensitivity/specificity) but slightly lower CA for *P. aeruginosa* (73%). CHROMagar COL‐APSE demonstrated acceptable sensitivity (92%) but low specificity (69%) in Enterobacterales. MicroScan had reduced specificity in *Enterobacterales* (87.80%). The CHROMAgar COL‐*APSE* efficiently identified the species with their unique colours but was the least specific (68.63%), with the highest ME in *Enterobacterales*. The rapid NP test provided rapid results within 4 h but showed a relatively high VME (7.84%), despite maintaining an acceptable sensitivity (92.16%) and specificity (96.08%). For laboratories with automated platforms, Vitek 2 remains optimal for colistin MIC testing; Sensititre and ComASP are suitable low‐cost BMD alternatives. The Rapid NP test provides a same‐day screen, but confirmatory MIC testing is advised. CHROMagar COL‐APSE should be used with a ≤ 1 CFU mL⁻¹ inoculum to minimise false resistance calls. Knowing the comparative performance of these different tests will assist in choosing the best test for every species, improving on efficient diagnosis and healthcare outcomes.

## Graphical abstract

Colistin‐resistance diagnostics that are efficient, affordable, easy to use, with quicker turnaround times are important for surveillance and control of resistance. The in‐house Rapid NP test has very major errors but is both affordable and fast, with 92.16% sensitivity and 96.00% specificity. In descending order, the Vitek 2, ComASP colistin, Sensititre, and MicroScan had high efficiencies and a turnaround time of 18–24 h. CHROMAgar COL‐*APSE* was more efficient in *A. baumannii* than *Enterobacterales*, with significant rates of major and very major errors.

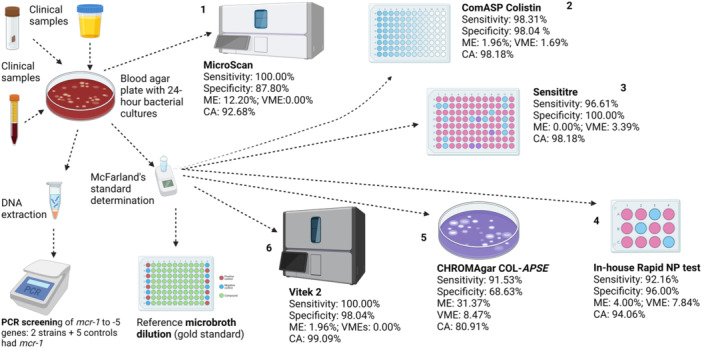

## 1 Introduction

Increasing use of carbapenems to treat multidrug‐resistant bacterial infections invariably led to the adoption of colistin as a last‐reserve antibiotic to counter infections that are resistant to carbapenems (Mmatli et al. 2020, 2022a, 2022c). Hence, bacteria that are resistant to colistin are increasingly being reported worldwide (Ramaloko and Osei Sekyere 2022; Lowe et al. 2022; Jayol et al. 2016). Resistance to colistin is mediated by several molecular mechanisms, including the mobile colistin resistance (*mcr*) gene, mutations in the PmrAB and PhoPQ two‐component systems, and mutation(s) in the MgrB regulator in *Klebsiella pneumoniae* (Mmatli et al. 2020, 2022c; Ramaloko and Osei Sekyere 2022; Osei Sekyere et al. 2021). These molecular mechanisms, alongside efflux hyperexpression and porin downregulation, and/or Ecr transmembrane protein, mediate phenotype colistin (polymyxin) resistance, and hetero‐resistance (Mmatli et al. 2020; Halaby et al. 2016; Poirel et al. 2017a).

To help clinicians easily detect and monitor colistin‐resistant infections, several diagnostic tests and assays, both commercial and noncommercial, have been designed and developed (Mmatli et al. 2022a; Leshaba et al. 2021; Osei Sekyere et al. 2020; Osei Sekyere 2019). Some of these tests (such as the BMD, ComASP™ Colistin, MicroScan, Sensititre and Vitek) are mainly MIC (minimum‐inhibitory concentration)‐based, measuring only the MICs of the isolates while others are non‐MIC—based, purely providing a binary result of resistant or sensitive (such as the CHROMagar COL‐*APSE*, and Rapid Polymyxin NP test) (Leshaba et al. 2021; Osei Sekyere et al. 2020; Osei Sekyere 2019). The Rapid Polymyxin NP test was designed by Nordmann Poirel to only detect colistin resistance in *Enterobacterales* and is therefore not useful for all Gram‐negative bacteria (Leshaba et al. 2021; Jayol et al. 2017). Several studies have evaluated the performance of these methods individually or in small combinations (Pfennigwerth et al. 2019; Osei Sekyere et al. 2020). Comparative evaluations across a broader panel of assays remain limited, particularly in resource‐constrained settings, where cost and accessibility influence test choice.

Recent multi‐centre studies have compared several colistin resistance diagnostics, highlighting both the strengths and context‐specific limitations of each (Dominguez et al. 2024; Silva et al. 2024). These comparative studies published since 2022, have examined subsets of these assays, yet none has evaluated all six head‐to‐head on a single, standardised isolate collection. A recent evaluation by Antony et al. compared Vitek 2, colistin broth‐disc elution, and reference broth microdilution in carbapenem‐resistant *Klebsiella pneumoniae*, reporting categorical agreement of 93.9% and very‐major‐error rates approaching 28.5% for Vitek 2 (Antony 2024). Silva et al. demonstrated excellent Rapid NP performance (sensitivity = 97.1%) but did not include non‐fermenters (Collar et al. 2024). A recent South‐East Asian multi‐centre study by Chung et al. found that CHROMagar COL‐APSE specificity dropped below 80% when a 0.5 McFarland inoculum was used (Chung et al. 2022). These reports underscore both the promise and context‐specific limitations of current diagnostics and highlight the need for consolidated, geographically diverse evaluations. Our study therefore provides a comprehensive, head‐to‐head assessment of six assays under ISO 20776‐1 conditions using South African clinical isolates.

Our study thus addresses this critical gap. We have evaluated some of the tests in a previous study. However, a follow‐up to our previous study is necessary to further validate the performance of these and other assays (Osei Sekyere et al. 2020). Herein, we used 142 Gram‐negative isolates and controls to evaluate the performance of six colistin resistance diagnostic tests: ComASP Colistin, CHROMagar COL‐*APSE*, Rapid NP test, Sensititre, Vitek 2, and the MicroScan. The BMD remains the gold standard for testing colistin MICs and resistance in bacteria (Leshaba et al. 2021) and was used as the reference standard in this study.

## 2 Methods

### 2.1 Demographics and Source of Clinical Specimen and Isolates

The evaluation study was conducted on a collection of 134 Gram‐negative clinical bacterial (GNB) isolates including *Enterobacterales* (*n* = 103), *Acinetobacter baumannii* (*n* = 21) and *Pseudomonas aeruginosa* (*n* = 10) that were collected from the National Health Laboratory Services, Tshwane Academic Division. Eight control strains were also included to make up to 142: *Escherichia coli* MC1, *E. coli* MC2, *E. coli* MC3, *Salmonella* group D MC4, *Salmonella* group D MC5, *E. coli* (mcr‐1) EMRC, *E. coli* ATCC 25922 (EATCC), *P. aeruginosa* ATCC 27853 (PATCC). Hence, there were 110 *Enterobacterales*, 21 *A. baumannii*, and 11 *P. aeruginosa* (Table S1.1)

Species identification was performed as part of routine laboratory testing using Vitek® 2 automated system (Biomerieux, France) (Table S1). Demographic data such as sex, age and sample source were retrieved from the NHLS TrakCare system (Table S1). The National Food Institute, Technical University of Denmark provided five *mcr*‐gene control strains (four *E. coli* with *mcr*‐1, *mcr*‐2, *mcr*‐3, and *mcr*‐4, as well as one *Salmonella* spp. with *mcr*‐5) for this study. Antimicrobial susceptibility testing also included one *Escherichia coli* ATCC 25922 and one *P. aeruginosa* ATCC 27853 (Table S1.2).

The labels of the isolates used in this article are known to only the researchers as the original labels were deidentified to maintain the patients' anonymity.

#### 2.1.1 Broth Microdilution

The reference MIC of each isolate was determined by manual broth microdilution (BMD) according to ISO standard 20776‐1 (International Organization for Standardization 2019). Colistin sulfate powder (Glentham Life Sciences, England) was diluted in cation‐adjusted Mueller Hinton broth (CAMHB) in untreated 96‐well microtiter polystyrene plates (Eppendorf, Germany). Dilutions to the MIC range 128–0.25 µg/mL were established. Colistin sensitivity results were interpreted according EUCAST breakpoints (susceptible ≤ 2 mg/L; resistant > 2 mg/L) (Table S1.3) (Osei Sekyere et al. 2020).

#### 2.1.2 Detection of *mcr*‐Genes

All isolates were screened for the presence of *mcr*‐1, *mcr*‐2, *mcr*‐3, *mcr*‐4, and *mcr*‐5 genes using conventional multiplex PCR as previously described by Rebelo et al. (2018). The details of the PCR procedure are described in our previous study (Mmatli et al. 2022a), and the full primer sequences and cycling conditions are detailed in Table S2. Briefly, a 25 µL reaction contained 1 × DreamTaq Green buffer, 2 mM MgCl₂, 200 µM each dNTP, 0.2 µM of each primer (Table S2) and 1 U Taq polymerase. Cycling conditions: 95°C 5 min; 35 cycles of 95°C 30 s, 58°C 30 s, 72°C 1 min; final extension 72°C 5 min. Amplicons were resolved on 1.5% agarose gels stained with GelRed.

Representative gel images could not be included as they are already published in another article (Mmatli et al. 2022b). Positive controls were reference strains harboring *mcr‐1* to *mcr‐5*; supplied by the kind courtesy of Professor Rene S. Hendriksen of the National Food Institute, Technical University of Denmark. *E. coli* ATCC 25922 served as a negative control.

#### 2.1.3 Vitek 2 System

Besides species identification, the Vitek was also used to determine the MICs of the isolates, using the manufacturer's protocols.

#### 2.1.4 Microscan

The MicroScan walkaway system (Beckman Coulter, South Africa) was used to determine the sensitivity of the species to colistin using the manufacturer's protocol. Briefly, an overnight culture grown on blood agar was immediately standardized using the the prompt inoculation system provided in the reagent packaging for MicroScan AST (antimicrobial sensitivity testing) and identification (ID) testing. The suspension was placed into the N66 and processed overnight in the MicroScan machine.

#### 2.1.5 ComASP™ Colistin

ComASP™ Colistin by Liofilchem (Roseto degli Abruzzi, Italy) is a compact panel containing freeze‐dried colistin which when diluted should result in two‐fold dilutions ranging from 0.25 to 16 µg/mL (Osei Sekyere et al. 2020; Carretto et al. 2018). The nonautomated BMD‐based assay allows for four samples to be tested on a single test. The manufacturer's instructions were followed to perform the ComASP™ Colistin test. A 0.5 McFarland suspension of the isolates was prepared in a solution of 250 mL saline and then diluted to 1:20 in saline (Gibco, Thermo Fisher Scientific, USA) to obtain solution A. Solution A (0.4 mL) was added to a tube containing 3.6 mL Mueller‐Hinton broth provided by the ComASP™ Colistin to obtain solution B. One hundred microliters of solution B were dispensed into each well and the panels were incubated (Vacutec, US) at 36°C ± 2°C for 16–20 h in ambient air. Results were read by visually analysing the plates for turbidity.

#### 2.1.6 ChromAgar Col‐*APSE*

Fresh 24‐h culture of each isolate was dissolved in saline and adjusted to 0.5 McFarland's standard. This suspension was spread on the ChromAgar Col‐*APSE* plate and incubated for 18–24 h. The manufacturer's protocol was used throughout this test (Osei Sekyere et al. 2020).

#### 2.1.7 Sensititre™

Sensititre™ (Thermo Fisher Scientific, USA) “FROL” colistin‐customised plates were used in this study. A 0.5 McFarland standard in saline was prepared for each isolate (Gibco, Thermo Fisher Scientific, USA). Ten microlitres of the 0.5 McFarland suspension was transferred into an 11 mL tube containing CAMHB with TES buffer for all non‐intrinsic *Enterobacterales*, *A. baumanni* and *P. aeruginosa* isolates. However, for *Proteus* species, *Providencia* spp., and *Morganella* morganii 1 μL was transferred into the 11 mL CAMHB tube. Each Sensititre™ plate constitutes of eight rows with 12 wells, each row allows for the testing of one isolate at different concentrations of colistin. Wells 1–11 contain dehydrated colistin at concentrations of 0.12–128 µg/mL, respectively. Well 12 represents a positive control and therefore does not contain colistin. Each well was inoculated with 50 μL of the bacterial broth suspension using a manual pipette. Each plate was then covered with an adhesive seal and incubated (Vacutec, US) at ±35°C in an aerobic environment. Results were read by visually analysing the plates for turbidity.

### 2.2 In‐House Rapid Polymyxin NP Test

Rapid Polymyxin NP test is based on the detection of glucose metabolism related to *Enterobacterales* growth in the presence of a defined concentration of colistin (Nordmann et al. 2016b). The formation of acid metabolites is shown by a colour change of a pH indicator (red phenol) in less than 4 h (Nordmann et al. 2016b; Mitton et al. 2019). The rapid NP test was performed as described by Nordmann et al. (2016b) (Jayol et al. 2016; Poirel 2017a; Jayol et al. 2018; Nordmann et al. 2016a). To prepare the NP test solution, a 6.25 g of CAMHB broth powder and 0.0125 g of phenol red powder was added to 225 mL of distilled water. The pH of the solution was then adjusted to 6.7 by adding 1‐N HCL in drops after which the solution was autoclaved. A 25 mL of 10% anhydrous D‐glucose solution that had been sterilised by filtration was added to the autoclaved CAMHB solution. Colistin stock solution (1280 µg/mL) was diluted to 200 µg/mL working solution by adding 1 mL of the stock solution to 5.4 mL CAMHB. To make colistin‐NP test solution, 100 µL of the colistin working solution (200 µg/mL) was added to 3900 µL NP test solution to get a final concentration of 5 µg/mL. The Rapid Polymyxin NP test was carried out in 96 well polystyrene plates (Eppendorf, Germany). A 150 µL of NP test solution alone without colistin was added to wells 1, 3, 5, 7, 9, and 11 of each row (A‐H) of the 96‐well microtiter plates. Wells 2, 4, 6, 8, 10, and 12 contained 150 µL of colistin‐NP test solution (5 µg/mL). *Proteus mirabilis* isolate from our collection was used as a resistant control and *Escherichia coli* ATCC 25922 was used as a susceptible control.

A 3.5 McFarland standard was prepared for each test isolates with sterile 0.85% NaCl solution and used within 15 of preparation. Each *Enterobacterales* isolate was inoculated (50 µL) in parallel into the two wells, one without colistin and one with colistin. Wells H7‐8 were inoculated 50 µL *P. mirabilis* and wells H9‐10 were inoculated with 50 µL *E. coli* ATCC 25922. Wells H11‐12 were inoculated with 50 µL of 0.85% NaCl solution. The microtiter plates were incubated for 3 h at 35°C ± 2, after 3 h of incubation, the isolates were checked for change in colour every 15 min until 4 h of incubation. The results were read visually where a change in colour of phenol red (orange to bright yellow) indicated growth.

### 2.3 Data Analysis

All six antimicrobial susceptibility testing methods were compared to the ISO standard 20776‐1 BMD. Colistin MIC results were interpreted using EUCAST's breakpoints (European Committee on Antimicrobial Susceptibility Testing, EUCAST 2024): *Enterobacterales* and *Acinetobacter baumannii* (susceptible ≤ 2 µg/mL; resistant, > 2 µg/mL); *Pseudomonas aeruginosa* (susceptible ≤ 4 µg/mL; resistant, > 4 µg/mL). For each test, the false positives (FP), false negatives (FN), true positives (TP), and true negatives (TN) were determined and used for downstream determination of the other performance indices: sensitivity, specificity, positive‐predictive value (PPV), negative‐predictive value (NPV) (Table S1). Sensitivity is the ability of the test to identify the presence of a disease or illness correctly. Specificity is the ability of the test to identify the absence of a disease or illness correctly (Table S1.2–S1.7).

We calculated the rates of essential agreement (EA), categorical agreement (CA), very major error (VME), and major error (ME) using already described methods (Osei Sekyere et al. 2020). Categorical agreement was defined as an agreement in the classification of susceptible or resistant between the evaluated test and the reference BMD. A very major error occurred when the tested method interpretation of an isolate was susceptible while the BMD interpretation was resistant for the same isolate. A major error occurred when the investigated method's interpretation was resistant, and the BMD interpretation was susceptible for the same isolate. Accuracy is the overall probability that a test is correctly classified. The sensitivity and specificity of each test were calculated as previously described (Osei Sekyere et al. 2020).

The isolates were divided into Enterobacterales, non‐fermenters (*A. baumannii* and *P. aeruginosa*), *A. baumannii*, and *P. aeruginosa*. The sensitivity, specificity, PPV, NPV, EA, CA, VME, and ME were determined separately for Enterobacterales and non‐fermenters (*A. baumannii* and *P. aeruginosa*) as well as for the individual *A. baumannii* and *P. aeruginosa* species (Table S1.8–S1.9).

## 3 Results

**Figure 1:**
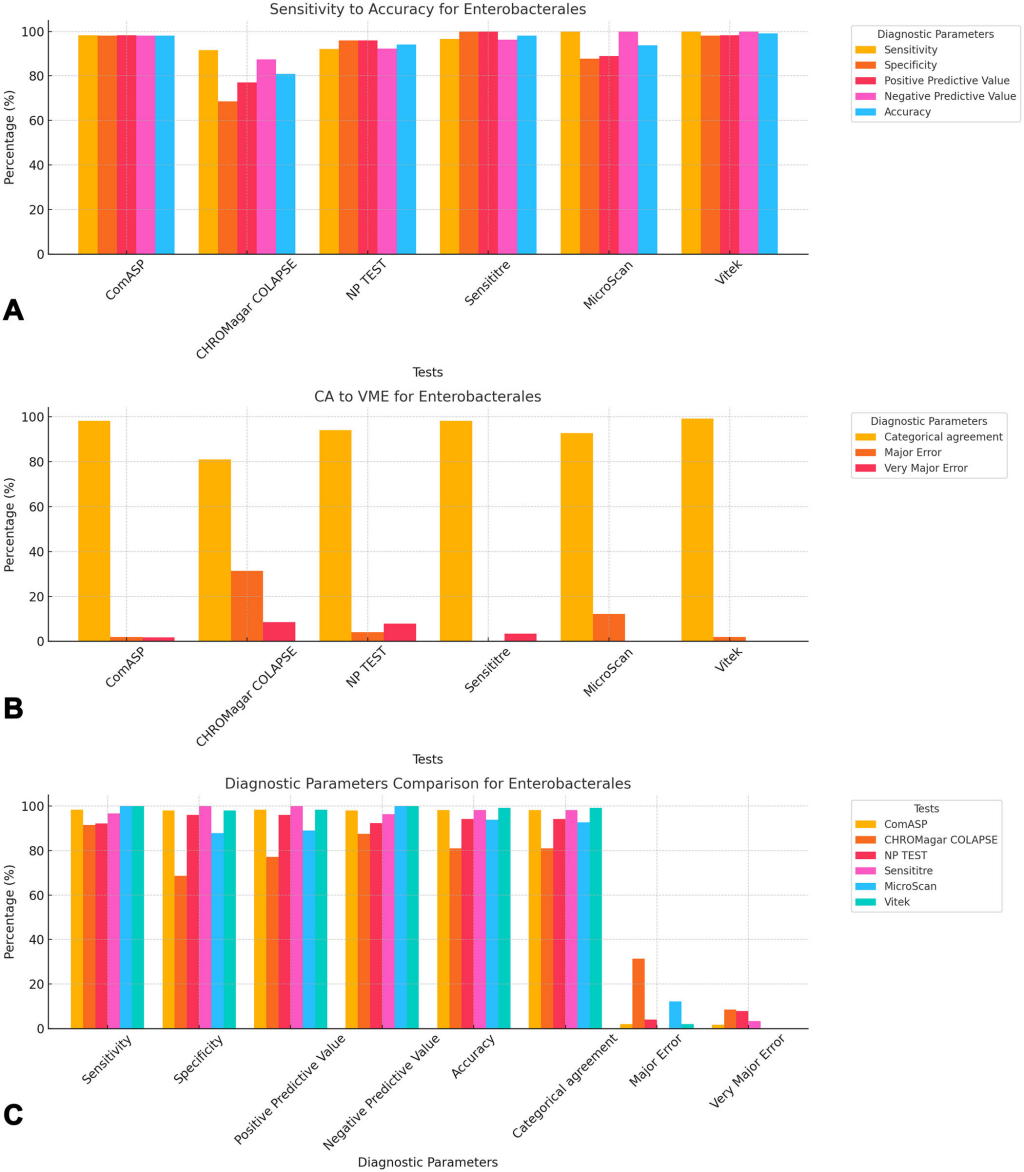
Diagnostic efficiency parameters of ComASP, CHROMagar COL‐APSE, NP test, MicroScan, and Vitek against Enterobacterales isolates. Sensitivity, Specificity, positive predictive value, negative predictive value, and accuracy are shown in plot A, categorical agreement, major error, and very major error are shown in plot B, and a comparison of all the measured diagnostic parameters across all the tests are shown in plot C. Panel A: Classical diagnostic indices—sensitivity (orange), specificity (red), positive‐predictive value (PPV, magenta), negative‐predictive value (NPV, cyan) and overall accuracy (blue). Panel B: Categorical agreement (CA, gold) versus the reference broth micro‐dilution (BMD) method and very major error (VME, dark red; false‐susceptible calls). Panel C: Radar‐style bar cluster summarising all parameters to enable rapid ranking. Vitek 2 achieved the highest CA (99.1%) with 0% VME; CHROMagar COL‐APSE showed the poorest specificity (68.6%) and the highest VME (8.5%). ME = major error; NP = Rapid Polymyxin NP test. Only Enterobacterales isolates were graphed in this figure; corresponding non‐fermenter data are shown in Figure 2.

**Figure 2:**
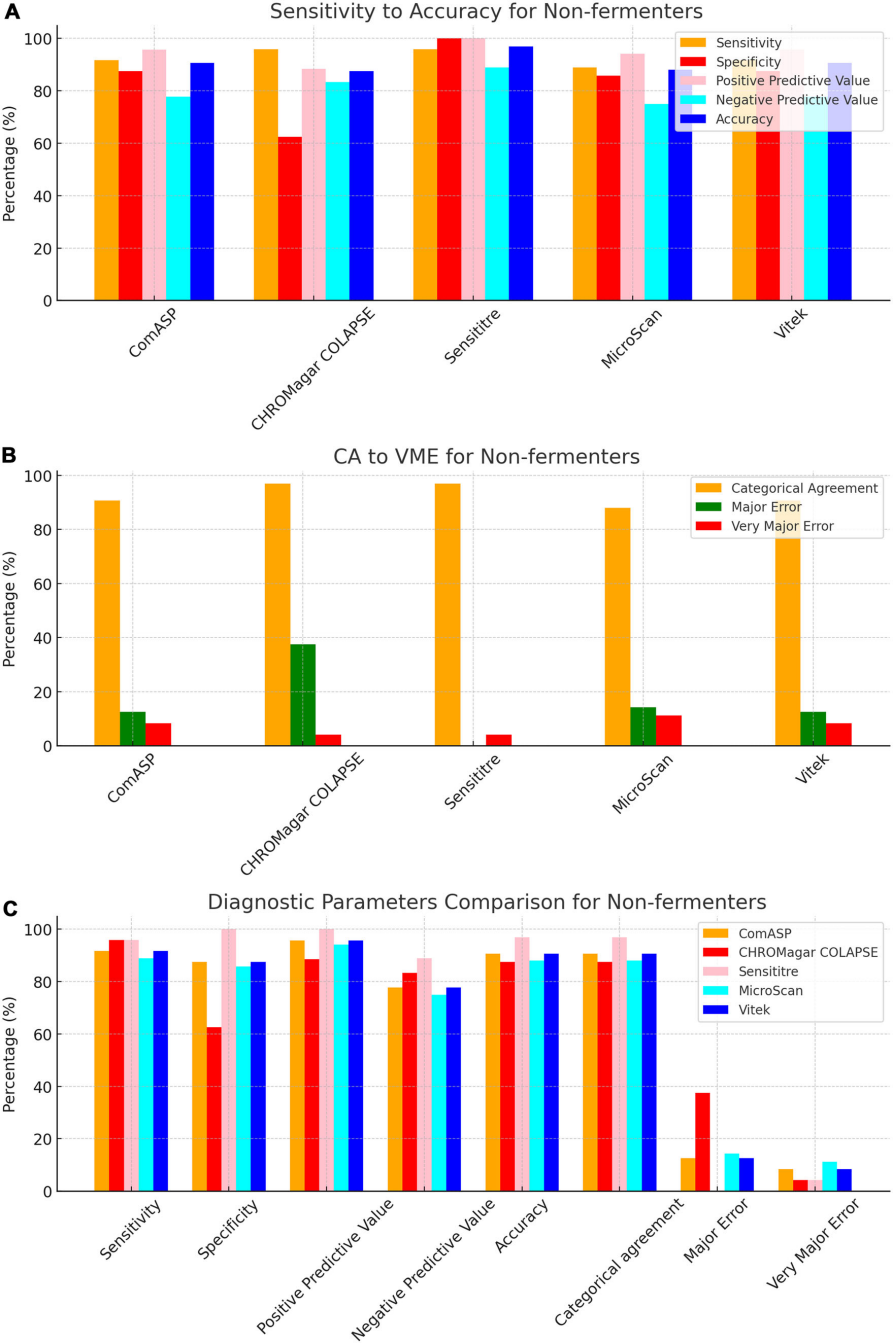
Diagnostic efficiency parameters of ComASP, CHROMagar COL‐APSE, MicroScan, and Vitek against non‐fermenters. Sensitivity, Specificity, positive predictive value, negative predictive value, and accuracy are shown in plot A, categorical agreement, major error, and very major error are shown in plot B, and a comparison of all the measured diagnostic parameters across all the tests are shown in plot C. Panel A: Sensitivity, specificity, PPV, NPV and accuracy (colour scheme as in Figure 1a). Sensititre retained ≥ 95% for all five metrics, while MicroScan lost specificity (74.0%). Panel B: CA and error profile. CHROMagar COL‐APSE recorded the highest ME (37.5%) but a moderate VME (4.2%). Panel C: Aggregate bar chart emphasising that Sensititre is the most reliable overall for non‐fermenters, whereas Vitek 2 under‐called A. baumannii resistance (VME 8.3%). Sample denominations: A. baumannii = 21; P. aeruginosa = 11.

**Figure 3:**
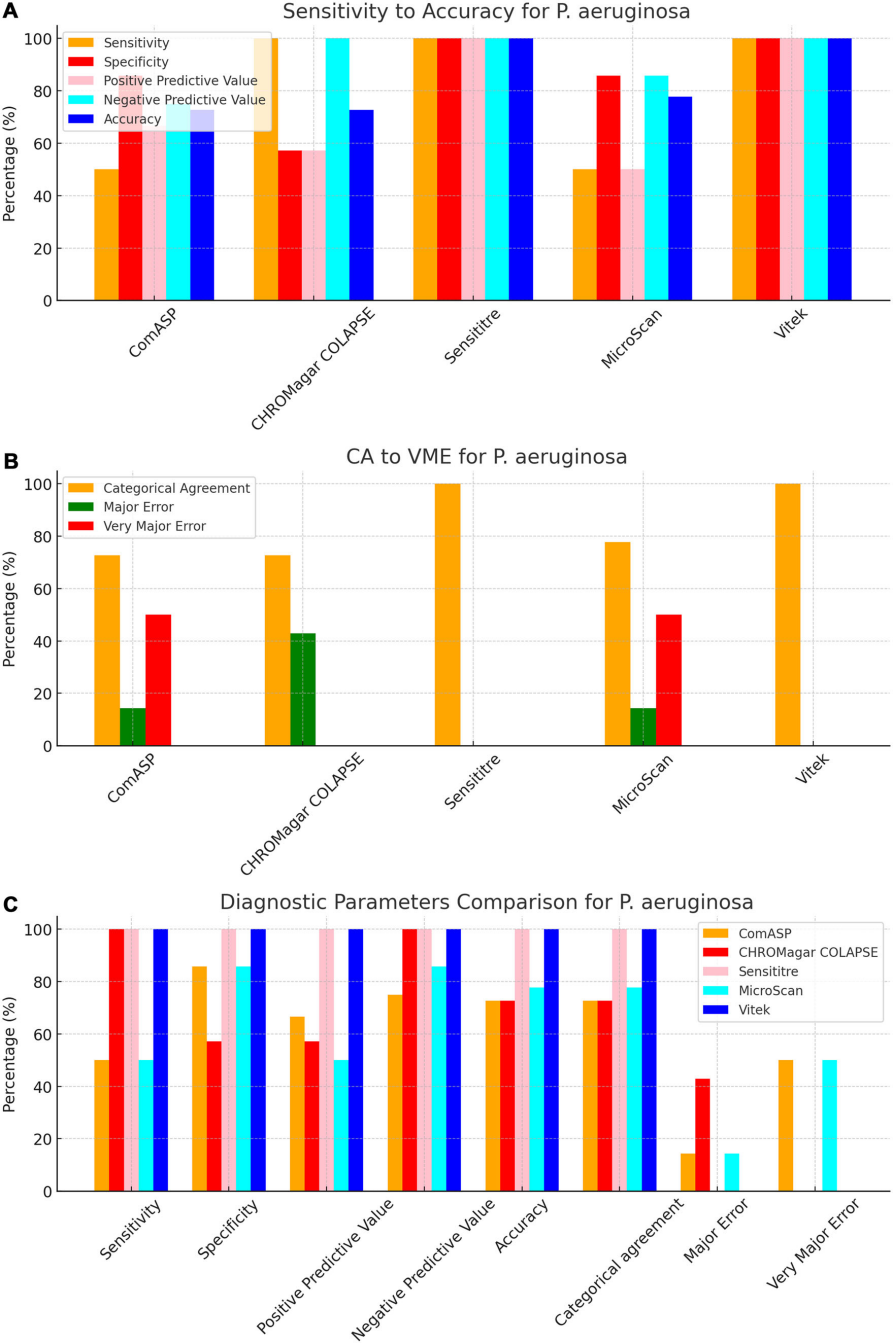
Diagnostic efficiency parameters of ComASP, CHROMagar COL‐APSE, MicroScan, and Vitek against P. aeruginosa. Sensitivity, Specificity, positive predictive value, negative predictive value, and accuracy are shown in plot A, categorical agreement, major error, and very major error are shown in plot B, and a comparison of all the measured diagnostic parameters across all the tests are shown in plot C. Panel A: Five key indices. Vitek 2, MicroScan, and Sensititre displayed perfect specificity (100%); only Vitek 2 and CHROMagar COL‐APSE achieved 100% sensitivity. Panel B: CA plus error categories. ComASP and MicroScan generated one VME each (50% of resistant isolates missed); CHROMagar COL‐APSE produced the highest ME (43%). Panel C: Full parameter overlay illustrating that Vitek 2 was the only assay with 0% errors and 100% in all diagnostic indices for P. aeruginosa in this cohort.

**Figure 4:**
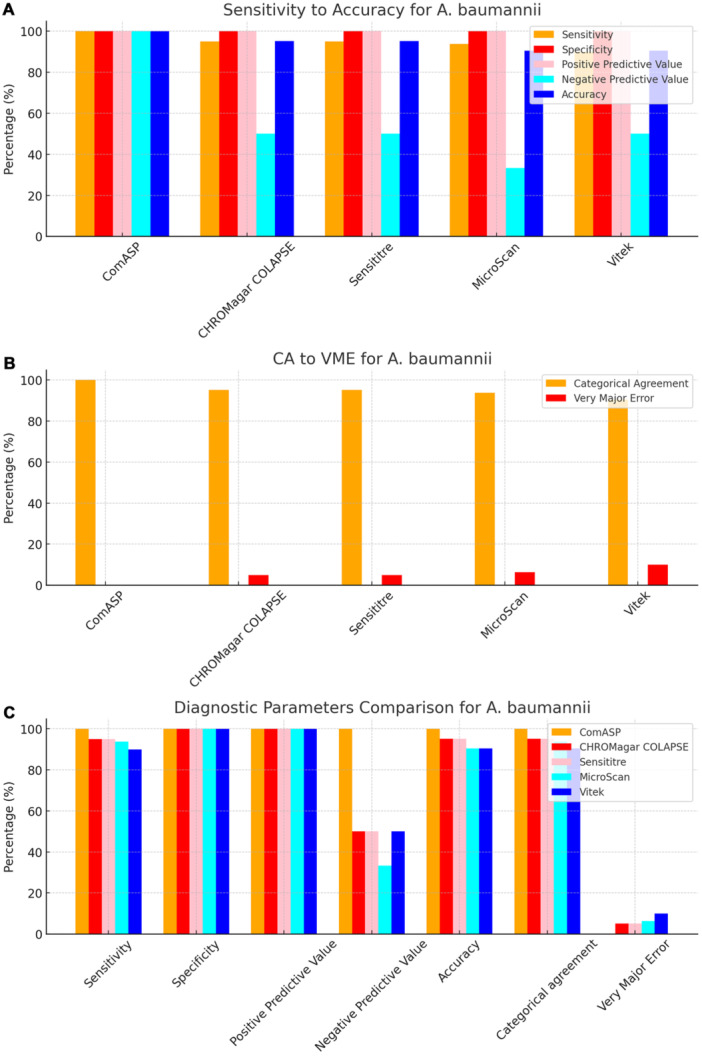
Diagnostic efficiency parameters of ComASP, CHROMagar COL‐APSE, MicroScan, and Vitek against A. baumannii. Panel A: Diagnostic indices. ComASP, Sensititre, and CHROMagar COL‐APSE all returned 100% sensitivity; Sensititre and CHROMagar COL‐APSE matched this with 100% specificity, whereas Vitek 2 lagged (90%). Panel B: CA and VME. Only CHROMagar COL‐APSE, Sensititre, and ComASP reached the CLSI/EUCAST target of ≥ 95% CA; Vitek 2 registered the highest VME (10%). Panel C: Composite comparison confirms ComASP as the sole assay with 100% scores across sensitivity, specificity, PPV, NPV, accuracy, and CA for this species.

**Figure 5:**
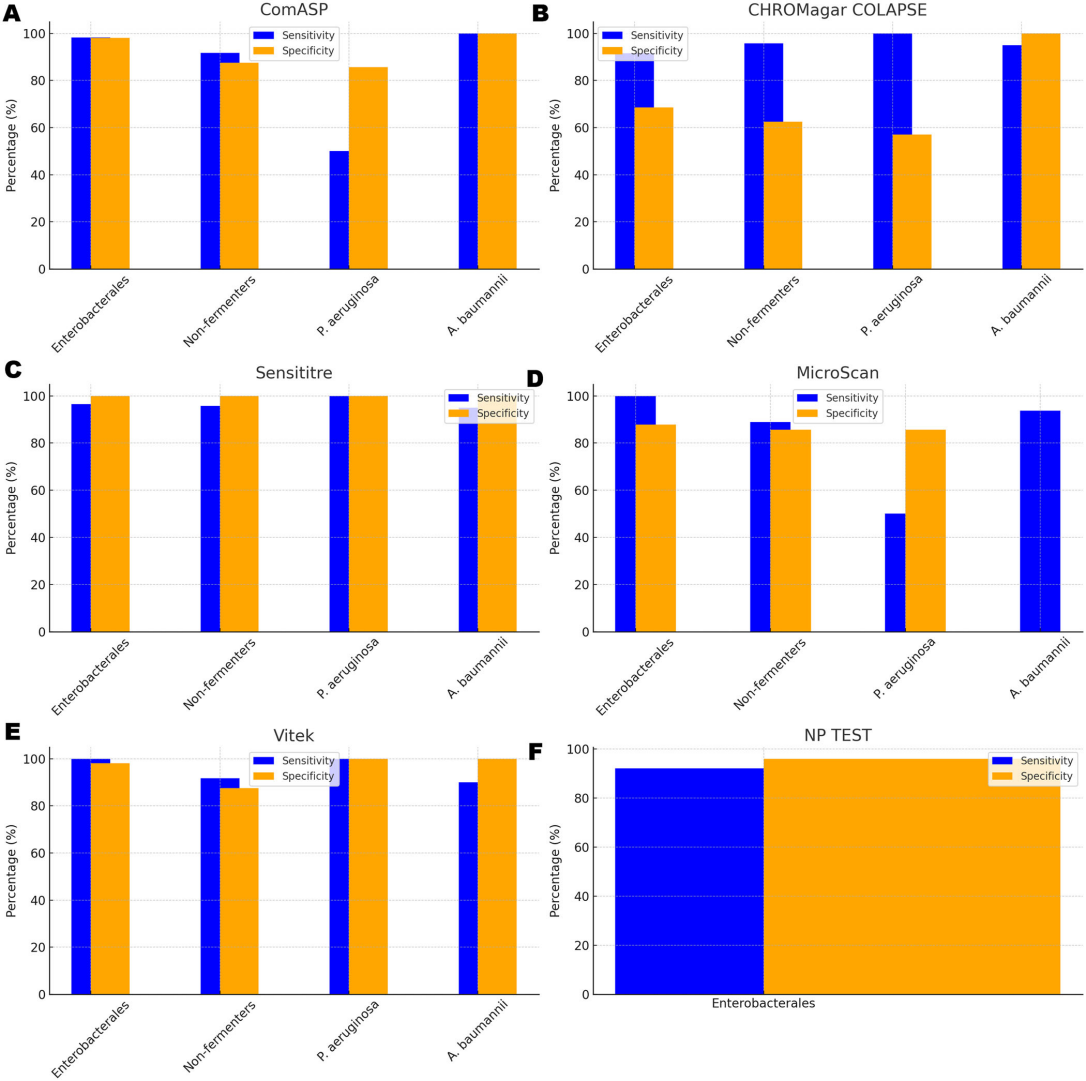
Sensitivity and specificity of ComASP, CHROMagar COL‐APSE, NP test, MicroScan, and Vitek across Enterobacterales and non‐fermenters. Each of the six plots shows one test, with sensitivity (blue bars) and specificity (yellow bars). The stronger performance of ComASP, Sensititre, and Vitek can be easily seen from these plots. Panel A (ComASP Colistin)—Blue bars: sensitivity; yellow bars: specificity for Enterobacterales, non‐fermenters, Pseudomonas aeruginosa, and Acinetobacter baumannii. ComASP maintains ≥ 96% sensitivity for Enterobacterales and A. baumannii but drops to 60% for P. aeruginosa (breakpoint proximity effect). Panel B (CHROMagar COL‐APSE)—Sensitivity is acceptable for all taxa ( ≥ 91%), whereas specificity plummets to 69% in Enterobacterales because of Klebsiella false‐positives; specificity remains < 60% for non‐fermenters. Panel C (Sensititre)—Shows near‐perfect balance: ≥ 96% sensitivity and ≥ 97% specificity for every group, confirming Sensititre as the most consistent commercial BMD kit across species. Panel D (MicroScan)—Retains 100% sensitivity in Enterobacterales but specificity is 88%; performance drops in non‐fermenters (sensitivity 89%, specificity 83%) and for P. aeruginosa (specificity 0% because all isolates in this cohort were resistant). Panel E (Vitek 2)—Displays the highest combined sensitivity/specificity for Enterobacterales (100%/98%) and P. aeruginosa (100%/100%). Slight under‐calling of A. baumannii resistance reduces sensitivity to 90%. Panel F (Rapid Polymyxin NP test)—Only Enterobacterales were tested: sensitivity 92%, specificity 96%. Bars for other taxa are not plotted because the assay is validated only for Enterobacterales. Abbreviations: Sens = sensitivity; Spec = specificity; NF = non‐fermenters (combined A. baumannii + P. aeruginosa). Error bars reflect ±95% confidence intervals.

**Figure 6:**
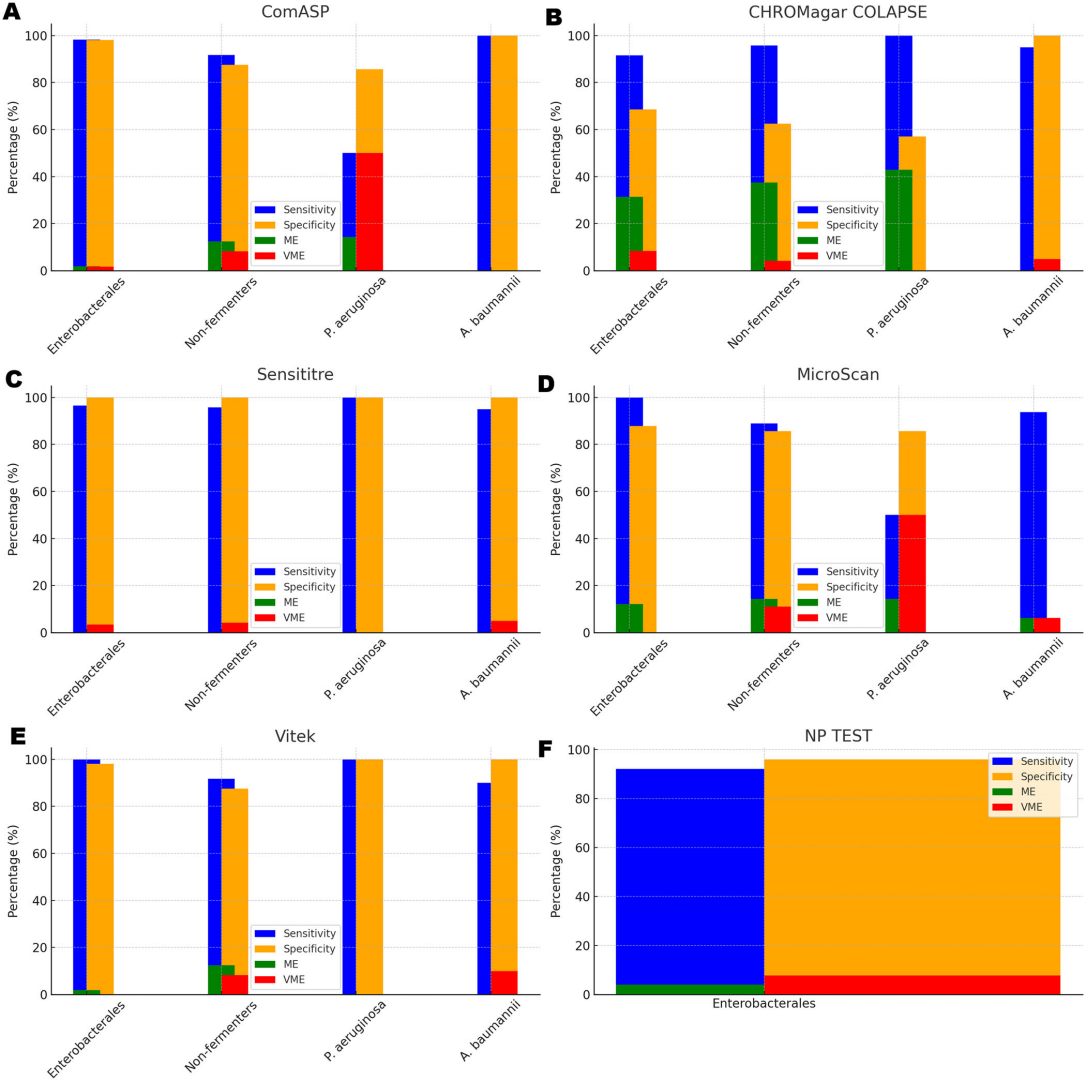
A bar chart showing the sensitivity (blue bar), specificity (yellow bar), major error (red bar), and very major error (green bar) of the six diagnostic tests against Enterobacterales and, non‐fermenters. The performance of each of the tests against these organisms can be easily seen from this plot. ComASP performed strongly against Enterobacterales and A. baumannii, Sensititre was strong across all isolates and Vitek was better with P. aeruginosa and Enterobacterales. Panel A (ComASP Colistin)—sensitivity and specificity remain high for Enterobacterales and A. baumannii; however, P. aeruginosa shows elevated very major error (VME = 33%) and major error (ME = 20%), highlighting breakpoint‐straddling MICs in this species. Panel B (CHROMagar COL‐APSE)—Exhibits the highest ME across Enterobacterales (31%) and non‐fermenters (38%). VME for P. aeruginosa is 0%, but specificity remains low. Panel C (Sensititre)—ME is negligible (0%), and VME is ≤ 5% in all taxa; overall, the only assay meeting CLSI acceptance criteria (≤ 1.5% VME for Enterobacterales) except for a single VME in A. baumannii. Panel D (MicroScan)—High ME (12%) and VME (0%) for Enterobacterales, but for P. aeruginosa both ME and VME surge (45% and 50%, respectively), driven by false‐susceptible calls at 4 µg mL^−^¹. Panel E (Vitek 2)—Only assay with 0% VME in Enterobacterales and P. aeruginosa; a 10% VME in A. baumannii stems from two isolates with MIC = 4 µg mL^−^¹ that were mis‐categorised as susceptible. Panel F (Rapid Polymyxin NP test, Enterobacterales only)—Sensitivity 92%, specificity 96%; ME 4%; VME 8%. Despite higher error rates than BMD‐based assays, its ≤ 4 h turnaround makes it a pragmatic first‐line screen. Colour code (all panels): Blue = sensitivity, gold = specificity, red = major error (false‐resistant), green = very major error (false‐susceptible). ME and VME bars are plotted on the same 0%–100% scale for direct visual comparison of risk. Error bars represent ±95% confidence intervals derived from binomial proportions.

To facilitate direct comparison across assays, performance metrics are summarised in Table 1 and illustrated in composite Figures 1, 2, 3, 4, 5, 6. For each organism group we present (i) overall diagnostic performance (sensitivity, specificity, PPV, NPV, accuracy), (ii) error profile (CA, ME, VME), and (iii) a radar plot of all parameters. The text below highlights key inter‐assay contrasts; full numeric values appear in Table 1 and Table S1.

**Table 1:** Diagnostic performance of six colistin‐resistance assays versus ISO 20776‐1 broth microdilution.

	ComASP (95% CI). *n* = 110	CHROMagar COLAPSE (95% CI). *n* = 110	NP^c^ TEST (95% CI). *n* = 101	Sensititre^b^ (95% CI). *n* = 110	MicroScan^b^ (95% CI) *n* = 82	Vitek^b^ (95% CI). *n* = 110
ENTEROBACTERALES						
Sensitivity	98.31% (90.91–99.96%)	91.53% (81.32%–97.19%)	92.16% (81.12%–97.82%)	96.61% (88.29%–99.59%)	100.00% (91.19%–100.00%)	100.00% (93.94%–100.00%)
Specificity	98.04% (89.55%– 99.95%)	68.63% (54.11%–80.89%)	96.00% (86.29%–99.51%)	100.00% (93.02%–100.00%)	87.80% (73.80%–95.92%)	98.04% (89.55%–99.95%)
Positive Predictive Value	98.31% (89.28%– 99.75%)	77.14% (69.06%–83.61%)	95.92% (85.77%–98.92%)	100.00% (93.73%–100.00%)	88.89% (77.87%–94.79%)	98.33% (89.44%–99.76%)
Negative Predictive Value	98.04% (87.74%– 99.71%)	87.50% (74.78%–94.29%)	92.31% (82.38%–96.85%)	96.23% (86.72%–99.01%)	100.00% (90.26%–100.00%)	100.00% (92.89%–100.00%)
Accuracy	98.18% (93.59%–99.78%)	80.91% (72.31%–87.78%)	94.06% (87.52%–97.79%)	98.18% (93.59%–99.78%)	93.83% (86.18%–97.97%)	99.09% (95.04%–99.98%)
Categorical agreement	108 (98.18%)	89 (80.91%)	95 (94.06%)	108 (98.18%)	76 (92.68%)	109 (99.09%)
Major error	1 (1.96%)	16 (31.37%)	2 (4.00%)	0 (0.00%)	5 (12.20%)	1 (1.96%)
Very major error	1 (1.69%)	5 (8.47%)	4 (7.84%)	2 (3.39%)	0 (0.00%)	0 (0.00%)
Turnaround time (hrs)	16–20	16–20	16–20	16–20	16–20	16–20
Non‐fermenters (*A. baumannii and P. aeruginosa*)						
Sensitivity	91.67% (73.00%–98.97%)	95.83% (78.88%–99.89%)		95.83% (78.88%–99.89%)	88.89% (65.29%–98.62%)	91.67% (73.00%–98.97%)
Specificity	87.50% (47.35%–99.68%)	62.50% (24.49%–91.48%)		100.00% (63.06%–100.00%)	85.71% (42.13%–99.64%)	87.50% (47.35%–99.68%)
Positive predictive value	95.65% (77.79%–99.28%)	88.46% (75.74%–94.96%)		100.00% (85.18%–100.00%)	94.12% (72.12%–99.00%)	95.65% (77.79%–99.28%)
Negative predictive value	77.78% (47.51%–93.12%)	83.33% (40.54%–97.35%)		88.89% (54.01%–98.20%)	75.00% (43.96%–91.98%)	77.78% (47.51%–93.12%)
Accuracy	90.62% (74.98%–98.02%)	87.50% (71.01%–96.49%)		96.88% (83.78%–99.92%)	88.00% (68.78%–97.45%)	90.62% (74.98%–98.02%)
Categorical agreement	29 (90.63%)	28 (87.50%)		31 (96.88%)	22 (88.00%)	29 (90.63%)
Major error	1 (12.50%)	3 (37.50%)		0 (0.00%)	1 (14.29%)	1 (12.50%)
Very major error	2 (8.33%)	1 (4.17%)		1 (4.17%)	2 (11.11%)	2 (8.33%)
Turnaround time (hrs)^a^	16–20	16–20	16–20	16–20	16–20	16–20
*P. aeruginosa*						
Sensitivity	50.00% (6.76%–93.24%)	100.00% (39.76%–100.00%)		100.00% (39.76%–100.00%)	50.00% (1.26%–98.74%)	100.00% (39.76%–100.00%)
Specificity	85.71% (42.13%–99.64%)	57.14% (18.41%–90.10%)		100.00% (59.04%–100.00%)	85.71% (42.13%–99.64%)	100.00% (59.04%–100.00%)
Positive predictive value	66.67% (20.28%–94.02%)	57.14% (36.18%–75.82%)		100.00% (39.76%–100.00%)	50.00% (9.25%–90.75%)	100.00% (39.76%–100.00%)
Negative predictive value	75.00% (51.82%–89.32%)	100.00% (39.76%–100.00%)		100.00% (59.04%–100.00%)	85.71% (59.22%–96.12%)	100.00% (59.04%–100.00%)
Accuracy	72.73% (39.03%–93.98%)	72.73% (39.03%–93.98%)		100.00% (71.51%–100.00%)	77.78% (39.99%–97.19%)	100.00% (71.51%–100.00%)
Categorical agreement	8 (72.72%)	8 (72.72%)		11 (100%)	7 (77.78%)	11 (100%)
Major error	1 (14.29%)	3 (42.86%)		0 (0.00%)	1 (14.29%)	0 (0.00%)
Very major error	2 (50.00%)	0 (0.00%)		0 (0.00%)	1 (50.00%)	0 (0.00%)
Turnaround time (hrs)	16–20	16–20	16–20	16–20	16–20	16–20
*A. baumannii*						
Sensitivity	100.00% (83.16%–100.00%)	95.00% (75.13%–99.87%)		95.00% (75.13%–99.87%)	93.75% 69.77%–99.84%	90.00% (68.30%–98.77%)
Specificity	100.00% (2.50%–100.00%)	100.00% (2.50%–100.00%)		100.00% (2.50%–100.00%)		100.00% (2.50%–100.00%)
Positive predictive value	100.00% (83.16%–100.00%)	100.00% (82.35%–100.00%)		100.00% (82.35%–100.00%)	100.00% (78.20%–100.00%)	100.00% (81.47%–100.00%)
Negative predictive value	100.00% (2.50%–100.00%)	50.00% (12.89%–87.11%)		50.00% (12.89%–87.11%)		33.33% (11.84%–65.06%)
Accuracy	100.00% (83.89%–100.00%)	95.24% (76.18%–99.88%)		95.24% (76.18%–99.88%)		90.48% (69.62%–98.83%)
Categorical agreement	21 (100%)	21 (100%)		20 (95.24%)	15 (93.75%)	19 (90.48%)
Major error	0 (0.00%)	0 (0.00%)		0 (0.00%)		0 (0.00%)
Very major error	0 (0.00%)	1 (5.00%)		1 (5.00%)	1 (6.25%)	2 (10.00%)
Turnaround time (hrs)^a^	16–20	16–20	16–20	16–20	16–20	16–20

*Note:* Comparative diagnostic performance of six colistin susceptibility testing methods ComASP, CHROMagar COL‐APSE, Rapid NP test, Sensititre, MicroScan, and Vitek, across Enterobacterales, *Pseudomonas aeruginosa*, and *Acinetobacter baumannii* isolates. For each method and bacterial group, performance metrics including sensitivity, specificity, positive predictive value, negative predictive value, accuracy, categorical agreement, major error, very major error, and turnaround time are presented with 95% confidence intervals where applicable. The sample size (*N*) for each test is indicated. Categorical agreement is reported as both a percentage and absolute number of correctly categorized isolates. Turnaround time is presented in hours (HRS).

Abbreviations: CA, categorical agreement; CI, confidence interval; ME, major error; NPV, negative predictive value; PPV, positive predictive value; VME, very major error.

^a^ Turn‐around time reflects incubation plus instrument processing.

^b^ EUCAST v14.0 break‐points: susceptible ≤ 2 µg mL^–^¹ for Enterobacterales and *A. baumannii*; ≤ 4 µg mL^–^¹ for *P. aeruginosa*.

^c^ NP = rapid polymyxin NP (Nordmann/Poirel) test.

### 3.1 Reference BMD

Reference MICs of all 142 isolates were determined by standard BMD, either in this study or as standard protocol by the National Health Laboratory Services Tshwane Academic Division. One *A. baumannii*, 51 *Enterobacterales*, and seven *P. aeruginosa* isolates were colistin‐susceptible with BMD, including one *E. coli* ATCC 25922 and a *P. aeruginosa* ATCC 27853 (Table 1). The colistin‐resistant isolates by BMD include 59 *Enterobacterales*, 20 *A. baumannii*, and four *P. aeruginosa*. Apart from the five *mcr* control strains, only two isolates, BB2 and EMRC, had *mcr*‐1 genes (Table 1). Isolate BB2 is an *A. baumannii* with a MIC of 32 µg/mL, while isolate EMRC is an *E. coli* with a MIC of 4 µg/mL. All eight intrinsically colistin‐resistant *Enterobacterales* (*Proteus mirabilis, Serratia marcescens, Morganella morganii, Proteus vulgaris, Providencia stuartii, and Ralstonia/Burkholderia pickettii*) had MICs ≥ 128 μg/mL (Table 1).

### 3.2 Commercial BMD Methods

All 142 isolates were tested on ComASP Colistin and Sensititre. ComASP Colistin and Sensititre both had a categorical agreement of 98.18% for *Enterobacterales* but differed for the non‐fermenters (Table 1). However, ComASP Colistin falsely detected one *K. pneumoniae* (1 µg/mL) and one *P. aeruginosa* (1 µg/mL) as colistin‐susceptible isolates, resulting in 14.29% and 12.50% MEs for *P. aeruginosa* and non‐fermenters, respectively. Sensititre accurately identified all colistin‐susceptible isolates. However, it failed to identify three colistin‐resistant isolates, therefore resulting in no MEs and 3.39%, 4.17%, and 5.00% VMEs for *Enterobacterales*, non‐fermenters, and *A. baumannii*, respectively. Most isolates that were not detected by Sensititre and ComASP Colistin had MICs close to the breakpoints (4–8 µg/mL). Only one isolate that was not detected by Sensititre™ had an MIC > 8 µg/mL, that is, a *K. pneumoniae* with an MIC of 32 µg/mL. ComASP™ Colistin had a sensitivity and specificity of 98.31% and 98.04%, and 91.67% and 87.50% for *Enterobacterales* and non‐fermenters whereas Sensititre™ had 96.61% and 100%, and 95.83% and 100% for *Enterobacterales* and non‐fermenters, respectively (Table 1; Figures 1, 2, 3, 4). Both tests fared better in terms of sensitivity and specificity against *A. baumannii* than against *P. aeruginosa* (Figures 1, 2, 3, 4):

Notably, ComASP fared better with *Enterobacterales* and *A. baumannii* with 98.31% and 100.00% sensitivity, 98.04% and 100.00% specificity, 98.18% and 100.00%, respectively. Sensititre was very sensitive and specific to *P. aeruginosa*, followed by *Enterobacterales*, and *A. baumannii* (Table 1; Figures 1, 2, 3, 4, 5).

Vitek had the highest sensitivity (100.00%) and the second or third highest specificity (98.04% among *Enterobacterales*. It has the same sensitivity and specificity as ComASP for the non‐fermenters, after Sensititre, which had the best results for the non‐fermenters (Table 1, Figures 1, 2, 3, 4, 5). Together with the MicroScan and Rapid NP Test, it had the best performance against *P. aeruginosa*. It also had the highest CA (99.09%), and one of the lowest VME (0.00%) among the six tests against *Enterobacterales* (Figure 6). The Microscan was equally highly sensitive (100.00%) but less specific (87.80%) against *Enterobacterales* compared to the MIC‐based tests. It had the least sensitivity and specificity among the non‐fermenters, particularly against *P. aeruginosa* (Table 1; Figures 1, 2, 3, 4, 5, 6). The MicroScan's MEs with both non‐fermenters and *Enterobacterales* (14.29% and 12.20% respectively) were only second to that of the CHROMAgar Col‐*APSE* (Figure 6) and its VMEs were 11.11% (non‐fermenters) and 0.00% (*Enterobacterales*).

### 3.3 CHROMAgar Col‐APSE

BMD identified 19 (16 *Enterobacterales* and three non‐fermenters) of all the isolates growing on the *CHROMagar* as colistin susceptible, resulting in 31.37% (*Enterobacterales*) and 37.50% (non‐fermenters) ME, and 68.63% (*Enterobacterales*) and 62.50% (non‐fermenters) specificity. Most of the MEs (14/19) were due to *Klebsiella* spp. The chromogenic media detected most colistin‐resistant isolates (*n* = 96), but it also produced a high percentage of VMEs of 8.47% and 4.17%, resulting in a sensitivity of 91.53% and 95.83%, respectively, for *Enterobacterales* and non‐fermenters. Chromagar was however very sensitive and specific against *A. baumannii* (Table 1; Figures 1, 2, 3, 4, 5, 6).

Differentiation of the isolated bacteria by the morphological appearance of their colonies was as described by the manufacturer (Abdul Momin et al. 2017). Metallic blue (*Klebsiella*, *Enterobacter*, and *Serratia* spp.), cream white (*Acinetobacter*, *Salmonella*, and *Pseudomonas* spp.), pink‐red (*E. coli*) colonies were observed (Figure 1) (Abdul Momin et al. 2017). Furthermore, *E. coli* could be distinguished as fermenting (blue with pink borders) and non‐fermenting strains (pink). Swarming was observed on the *P. mirabilis*‐inoculated plate because the plate was not supplemented with p‐nitrophenyl glycerol as recommended by Abdul Momin et al. (2017) (Figure 1) (Abdul Momin et al. 2017).

### 3.4 In‐House Rapid NP Test

Only 101/142 of the isolates, which were all *Enterobacterales*, were included in this test. This included 51 colistin‐susceptible and 50 non‐intrinsically colistin‐resistant *Enterobacterales* isolates. The sensitivity and specificity of the in‐house Rapid polymyxin NP test were 92.16% and 96.00% respectively. Colistin‐resistant isolates that were not detected include one *Salmonella* spp., two *K. pneumoniae* and one *E. coli*, with MICs ranging from 4 to 128 µg/mL. Two of the false‐resistant isolates, also grew on CHROMAgar Col‐*APSE* (Table S1). In this study, most results were positive within 2 h of incubation. Six isolates, however, were not detectable after 3 h of incubation but were positive after 4 h. Among the slow‐growing isolates were four *Salmonella* spp., one *E. coli*, and one *E. cloacae*.

## 4 Discussion

The proliferation of colistin resistance among GNB has necessitated the development of better diagnostics to quickly and efficiently detect colistin‐resistant organisms to control their spread (Mmatli et al. 2020, 2022c; Ramaloko and Osei Sekyere 2022; Leshaba et al. 2021; Osei Sekyere et al. 2020). This study evaluated six antimicrobial susceptibility tests that were developed to detect colistin‐resistance with efficiency. Broth microdilution is currently the golden standard for colistin susceptibility testing; however, it is considered tedious and difficult (Leshaba et al. 2021). Vitek 2, ComASP™ Colistin, and MicroScan had the best detection (sensitivity) of colistin‐resistant isolates (Table 1). When compared with Sensititre™, CHROMagar Col‐*APSE*, the Rapid Polymyxin NP test, ComASP™ Colistin, and MicroScan, Vitek 2 produced the least VMEs at 0.00% for *P. aeruginosa* and *Enterobacterales;* it performed poorly with *A. baumannii*. This was followed by the MicroScan and ComASP, with VMEs of 11.11% versus 8.36% and 0.00% versus 1.69%, respectively, for non‐fermenters and *Enterobacterales* (Table 1; Figures 1, 2, 3, 4, 5, 6).

Our findings align with previous studies highlighting that Vitek 2 generally provides robust colistin resistance detection (Osei Sekyere et al. 2020). Contrastingly, CHROMagar COL‐APSE's poor specificity and high error rates in our study echo results observed by Ali et al. (2022), attributed to inoculum concentration variability. Rapid NP test's sensitivity (92%) fell below reported rates of ≥ 97% from studies such as Nordmann et al. (2016b) and Germ et al. (2021) (Poirel and Nordmann 2015; Germ et al. 2021; Poirel 2017b). These discrepancies emphasize the importance of standardizing bacterial inoculum and procedure adherence to optimize test reliability.

Therefore, the Vitek 2, which is common in many well‐resourced routine diagnostic microbiology laboratories, is very efficient in detecting colistin resistance albeit its lower sensitivity towards *A. baumannii* should be considered when using MICs from Vitek 2. Moreover, its initial cost, long turnaround time, and skill required to operate it make it inaccessible to low‐resourced laboratories. Although the MicroScan is also highly sensitive with low VMEs (particularly among *Enterobacterales*), its lower specificity and high ME, 18–24‐h turnaround time, operating skill required, and initial cost are also major disadvantages for low‐resourced laboratories (Leshaba et al. 2021; Osei Sekyere et al. 2020; Osei Sekyere 2019). The ComASP and Sensititre are two affordable commercial kits that can be adopted in low‐resourced laboratories for colistin resistance diagnosis. The Sensititre had 100% specificity and > 95.83% sensitivity for all isolates, making it comparable to the ComASP and just a little lower than the Vitek among all the six tests. Hence, in terms of cost and required skill, the Sensititre and ComASP are good alternatives for determining the colistin MICs and resistance in low‐resourced laboratories.

In this study, CHROMAgar Col‐*APSE* produced the highest rate of MEs and VME (except with *A. baumannii*), second only to that of the in‐house Rapid NP test (Table 1 and Table S1), particularly for *Klebsiella* spp. Although the specificity recorded in this study and that of Osei Sekyere et al (at 66.67%) agree, two other studies recorded a significantly higher specificity of ≥ 97% (Osei Sekyere et al. 2020; Abdul Momin et al. 2017; Ali et al. 2022). These discrepancies could be attributed to the different bacterial inoculum concentrations used. Ali et al demonstrated that when a 0.5 McFarland standard inoculum was used, colistin‐susceptible isolates were able to grow on the media (Ali et al. 2022), whereas the same isolates were inhibited when the inoculum was diluted to a density of 1 × $10^{5}$ CFU/mL. Therefore, the high rate of MEs in this present study could be due to the inoculum used being a 0.5 McFarland standard (1.5 × $10^{8}$ CFU).

The in‐house Rapid Polymyxin NP test was expected to have the best performance for *Enterobacterales*. Even though the test was designed specifically for colistin susceptibility testing on *Enterobacterales*, its performance was inferior to that of commercial BMD methods (Tables 1 and S1; Figure 6). Compared with other studies that have achieved a sensitivity of ≥ 97%, the performance of the in‐house Rapid Polymyxin in this study (~ 92%) is slightly poor (Nordmann et al. 2016a; Mitton et al. 2019; Germ et al. 2021). The CA of all the tests evaluated in this study except for CHROMAgar Col‐*APSE*, is within the recommended standard (≥ 90%) for antimicrobial susceptibility testing systems. Nevertheless, except for Vitek 2 and MicroScan, all the other evaluated tests had an unacceptable VME rate of ≥ 1.5% in only *Enterobacterales;* Vitek and Sensititre were within range for *P. aeruginosa* while ComASP was within range for *A. baumannii*. Only Sensititre had an acceptable rate of MEs (0.00%) when all isolates were considered, but ComASP Colistin's ME improved when only *Enterobacterales* were examined (Tables 1 and S1; Figures 1, 2, 3, 4, 5, 6).

Our data confirm the consistently high VME risk of CHROMagar COL‐APSE when a 0.5 McFarland inoculum is used, echoing findings by Chung et al. and Chung et al. (2022); Ali et al. (2022). Reducing inoculum to ≤ 1 CFU mL^−^¹ improves specificity to > 95% but compromises ease‐of‐use—an important consideration for routine diagnostics.

In contrast, the Rapid NP test produced same‐day results with acceptable CA (94%) but VME of 7.8%. Although Germ et al. (2021) reported VME ≤ 1% using commercial reagents, their assay cost is prohibitive for many low‐resource settings. Our in‐house formulation costs < US$1 per isolate, making it a viable screen where confirmatory MIC testing is feasible. The combined workflow we propose—Rapid NP screening followed by Sensititre or ComASP confirmation—minimises both expense and diagnostic delay.

Therefore, for low‐resourced laboratories and research institutions, the in‐house Rapid NP test is a better option in terms of turnaround time, cost, and efficiency than the CHROMAgar COL‐*APSE*, except that the latter has advantages of easy use and species identification through its chromogenic compounds. In terms of cost and turnaround time, the CHROMAgar is comparable to the two other commercial MIC tests: Sensititre and ComASP. Nevertheless, the higher efficiency of the former two and their ability to provide actual MICs make them recommendable. Yet, CHROMAgar is easier to use than these two MIC tests.

## 5 Conclusion

This study discovered that commercial BMD methods had the best overall performance. In the absence of resources to purchase the Vitek 2 or the MicroScan, we recommend the ComASP Colistin and Sensititre as potential alternative MIC tests for routine colistin antimicrobial susceptibility testing in clinical laboratories. However, isolates with MICs close to the breakpoint may be misinterpreted by commercial BMD tests. As the in‐house Rapid NP test had the quickest turnaround time, we recommend it for colistin susceptibility testing on *Enterobacterales*, particularly in laboratories with limited resources and labour skill. Notably, CHROMAgar Col‐APSE produced the most errors; with colistin being a last‐reserve antibiotic, minimal error is critical. Hence, the use of CHROMAgar Col‐*APSE* should be validated further, possibly with lower inoculum. Notably, all the tests were able to detect colistin resistance in *mcr‐*positive strains.

## Author Contributions

**Tumisho Mmatumelo Seipei Leshaba:** investigation, validation, data curation. **Masego Mmatli:** investigation, validation, methodology, resources. **Nontombi Marylucy Mbelle:** funding acquisition, project administration, data curation. **John Osei Sekyere:** conceptualization, investigation, funding acquisition, writing – original draft, validation, visualization, writing – review and editing, software, formal analysis, project administration, data curation, supervision, resources. All authors reviewed the final version of the manuscript.

## Ethics Statement

The study was approved by the Research Ethics Committee, Faculty of Health Sciences, University of Pretoria, under reference number 550/2020. The study complied with the ICH‐GCP guidelines and the Helsinki declaration.

## Conflicts of Interest

The authors declare no conflicts of interest.

## Supporting information


**Table S1.** Demographics, species, complete antibiotic resistance results data, and minimum inhibitory concentrations (MICs) of the isolates used in this study. This dataset comprises of nine different Tables labelled Table S1.1 to S1.9.

**Table S2. Primer sets used for conventional multiplex PCR detection of** ***mcr‐1*** **to** ***mcr‐5*** **genes**. Columns: (i) Target gene, (ii) Primer name, (iii) Oligonucleotide sequence (5' → 3'), (iv) Amplicon size (bp), and (v) Reference (Rebelo et al. 2018; Euro Surveill. 23:e006682). All primers were synthesised by Inqaba Biotechnical Industries (Pretoria, South Africa) and validated in singleplex prior to multiplex assembly. PCR cycling conditions followed Rebelo et al. with 30 cycles and an annealing temperature of 58°C. Amplicons were visualised on a 1.5 % agarose gel stained with GelRed. Positive controls: *E. coli* strains carrying *mcr‐1* to *mcr‐5* supplied by the National Food Institute, Technical University of Denmark.

## Data Availability

The data that supports the findings of this study are available in the supporting material of this article.
