# Supplementary material for: Evaluation of Six Commercial and Noncommercial Colistin Resistance Diagnostics
Source: Microbiologyopen. 2025 Jun 29;14(4):e70029. doi: 10.1002/mbo3.70029 (PMC12206951; doi:10.1002/mbo3.70029)
Supplement: Supplementary file 2 — Table S2. Primer sets used for conventional multiplex PCR detection of mcr‐1 to mcr‐5 genes. Columns: (i) Target gene, (ii) Primer name, (iii) Oligonucleotide sequence (5' → 3'), (iv) Amplicon size (bp), and (v) Reference (Rebelo et al. 2018; Euro Surveill. 23:e006682). All primers were synthesised by Inqaba Biotechnical Industries (Pretoria, South Africa) and validated in singleplex prior to multiplex assembly. PCR cycling conditions followed Rebelo et al. with 30 cycles and an annealing temperature of 58°C. Amplicons were visualised on a 1.5 % agarose gel stained with GelRed. Positive controls: E. coli strains carrying mcr‐1 to mcr‐5 supplied by the National Food Institute, Technical University of Denmark. [file MBO3-14-e70029-s001.dot]

| **Target** | **Primer (5'→3')** | **Size (bp)** | **Reference** |
| --- | --- | --- | --- |
| *mcr-1* F | GCT GAG CGT TTT CCT TTC T | 309 | 15 |
| *mcr-1* R | AGT CCA GCC TTT TCC TTT T |  |  |
| *mcr-2* F | AAT GGT CTC TTG GCT CCT | 567 | 15 |
| *mcr-2* R | TGC CAC AGC TTC TGA ACC |  |  |
| *mcr-3* F | ATC ATT TCT GGC GCA GTC | 218 | 15 |
| *mcr-3* R | AGC AGA GAA GCC ACT CAC |  |  |
| *mcr-4* F | TGC ATG CGC TCA AGA TTC | 428 | 15 |
| *mcr-4* R | CAG CGC GAA ATA AGC ACC |  |  |
| *mcr-5* F | TAC TGG GTT GGA GCT GTT | 164 | 15 |
| *mcr-5* R | TTA AGA GCC AGC ATA GCA |  |  |
